# Swarms of chemically modified antiviral siRNA targeting herpes simplex virus infection in human corneal epithelial cells

**DOI:** 10.1371/journal.ppat.1010688

**Published:** 2022-07-06

**Authors:** Kiira Kalke, Liisa M. Lund, Marie C. Nyman, Alesia A. Levanova, Arto Urtti, Minna M. Poranen, Veijo Hukkanen, Henrik Paavilainen

**Affiliations:** 1 Institute of Biomedicine, University of Turku, Turku, Finland; 2 Molecular and Integrative Biosciences Research Programme, Faculty of Biological and Environmental Sciences, University of Helsinki, Helsinki, Finland; 3 School of Pharmacy, University of Eastern Finland, Kuopio, Finland; 4 Faculty of Pharmacy, University of Helsinki, Helsinki, Finland; University of Wisconsin-Madison, UNITED STATES

## Abstract

Herpes simplex virus type 1 (HSV-1) is a common virus of mankind and HSV-1 infections are a significant cause of blindness. The current antiviral treatment of herpes infection relies on acyclovir and related compounds. However, acyclovir resistance emerges especially in the long term prophylactic treatment that is required for prevention of recurrent herpes keratitis. Earlier we have established antiviral siRNA swarms, targeting sequences of essential genes of HSV, as effective means of silencing the replication of HSV *in vitro* or *in vivo*. In this study, we show the antiviral efficacy of 2´-fluoro modified antiviral siRNA swarms against HSV-1 in human corneal epithelial cells (HCE). We studied HCE for innate immunity responses to HSV-1, to immunostimulatory cytotoxic double stranded RNA, and to the antiviral siRNA swarms, with or without a viral challenge. The panel of studied innate responses included interferon beta, lambda 1, interferon stimulated gene 54, human myxovirus resistance protein A, human myxovirus resistance protein B, toll-like receptor 3 and interferon kappa. Our results demonstrated that HCE cells are a suitable model to study antiviral RNAi efficacy and safety *in vitro*. In HCE cells, the antiviral siRNA swarms targeting the HSV UL29 gene and harboring 2´-fluoro modifications, were well tolerated, induced only modest innate immunity responses, and were highly antiviral with more than 99% inhibition of viral release. The antiviral effect of the 2’-fluoro modified swarm was more apparent than that of the unmodified antiviral siRNA swarm. Our results encourage further research *in vitro* and *in vivo* on antiviral siRNA swarm therapy of corneal HSV infection, especially with modified siRNA swarms.

## Author summary

Herpes simplex virus type 1 (HSV-1) is a common virus carried approximately by half of the global population. Though it is mostly known by causing cold sores, it also causes herpes keratitis, which is the leading cause of infectious blindness in the world. The treatment for herpes keratitis and other severe disease forms of herpes infection is insufficient, as resistant variants arise upon long-term prophylactic treatments. We have earlier developed an anti-HSV siRNA swarm, which has proven safe and effective in many cell types, in animal models, and against variants resistant to current first-in-line treatment. Most recently, we added modifications to the anti-HSV siRNA swarm, which increased its efficacy and stability. In this study, we show the efficacy and safety of the modified anti-HSV siRNA swarm in a cell line representing the treatment target tissue in herpes keratitis. Our results show that our modified anti-HSV siRNA swarm is a possibility for future therapy for herpes keratitis. The results encourage further research in an animal model of herpes keratitis in order to uncover the potential of our modified anti-HSV siRNA swarm.

## Introduction

Herpes simplex virus type 1 (HSV-1) is a common human pathogen causing many severe diseases, including herpes simplex keratitis (HSK), the leading cause of infectious blindness or severe visual impairment in developed countries [1,2]. Prophylactic chemotherapy is usually accomplished using acyclovir (ACV), or related compounds, to prevent potentially severe recurrences of HSV-1 [1,2]. Adversely, prophylactic treatment with ACV, especially when long-term, predisposes for emergence of ACV-resistant viral strains [3,4]. As a majority of available HSV treatments have a mechanism of action similar to ACV, ACV-resistant strains causing untreatable disease exacerbations may emerge. Moreover, the lack of vaccine and curative treatment for the elimination of latent HSV-1 infection underlines the unmet medical need for novel antivirals for the treatment of ACV-resistant HSK.

We have previously described the use of antiviral small interfering (si)RNA swarms for treatment of HSV-1 infection both *in vitro* [5–10] and *in vivo* [11]. The most potent siRNA swarm targets a conserved, 653-bp-long sequence in the essential *UL29* gene of HSV-1 [7]. Recently, we have shown the efficacy of UL29 siRNA swarm against ACV-resistant HSV-1 and against various circulating Finnish HSV-1 strains, independent of their varying ACV sensitivity [9]. Moreover, we discovered, that the antiviral efficacy of the UL29 siRNA swarm increases 1000-fold when cells representing nervous system are treated with 2´-fluoro modified siRNA swarms instead of a canonical siRNA swarm [8]. The most promising 2´-fluoro modified siRNA swarm included the modification in adenosines.

In the current study, our aim was to characterize whether 2’-fluoro-adenosine modified or unmodified UL29 siRNA swarms can prevent HSV-1 infection in a corneal epithelial cell line. We assessed the responses of host innate immunity during siRNA swarm treatment, with and without the viral challenge, which has not, to this extent, been done before. We used an immortalized human corneal epithelial cell line (HCE), which has not, to our knowledge, been used in any antiviral RNAi studies before. Therefore, we carefully determined the responses of the cell line to HSV-1 infection, and to transfection with a known cytotoxic dsRNA separately. The panel of studied innate responses included interferon beta (IFN-β), lambda 1 (IFN-λ1), interferon stimulated gene 54 (ISG54), human myxovirus resistance protein A (MxA), human myxovirus resistance protein B (MxB), toll-like receptor 3 (TLR3), and interferon kappa (IFN-κ), of which MxA, MxB, and IFN-κ were presented for the first time in this context. The HCE cell line has before shown suitable for assessing ocular drug pharmacokinetics [12–14] and toxicity [15], and is compatible with liposomal transfection [16], suggesting for high translationality of the obtained results.

Our results show that HCE cells are an appropriate cell line for determining the efficacy and tolerability of antiviral RNA interference (RNAi) *in vitro*. This is demonstrated by the capability of HCE cells to support HSV-1 infection and exhibit type I and type III innate responses upon treatment with a known immunostimulatory double-stranded (ds)RNA. Furthermore, we show that RNAi using UL29 siRNA swarms is sequence specific, highly effective against HSV-1, and only induces minimal innate immunity responses, independent of the modifications in the siRNA swarm. Our results indicate that adenosine modified siRNA swarms are slightly more antiviral than their unmodified counterparts also in HCE cells. The results encourage for further research on antiviral siRNA swarm therapy of infected cornea, especially with modified siRNA swarms.

## Methods

### Human corneal epithelial cells

An immortalized human corneal epithelial (HCE) cell line was used as a model for the target tissue of antiviral HSK treatment. Unlike in previous studies [12–16], we used the cells as a monolayer culture allowing for reproducible testing with high capacity in multi-well plates [17]. HCE cells were maintained in Dulbecco’s Modifed Eagle Medium (DMEM) with L-Glucose (4.5 g/L) (Lonza, cat. BE12-709F) supplemented with 7% Fetal Bovine Serum (FBS) (Serana, cat. S-FBS-AU-015). The cells were plated one day prior to initiation of the assays on 96-well plates (Corning, cat. 3595) to reach a confluency of 40–50% on the starting day of the experiment.

### RNA production

The RNAs used in this study are listed in Table 1. SiRNAs were cleaved from an enzymatically synthesized target gene-specific long dsRNA using recombinant *Giardia intestinalis* Dicer. The antiviral siRNA swarms used in this study targeted a 653 bp sequence of the essential *UL29* gene of HSV-1. The UL29-targeted siRNA swarms were either unmodified or had 2’-fluoro-modifications in their ribose backbone. The 2’-fluoro-modifications were introduced into the RNA backbone during the synthesis of the long target-specific dsRNAs by replacing the canonical ATP with corresponding 2′-F-ATP either fully or partly. The resulting siRNAs are referred to here as 100% F-A or 10% F-A, respectively. As controls for any non-specific antiviral effects, two different siRNA swarms, without homology to any genes of wild type (wt) HSV or of human, were used. One was a GFP-targeted siRNA swarm [7], referred in this paper as transgene specific control, resulting in activation of RNAi as the HSV strain used in the study encodes a nonessential GFP transgene, and the other was a non-HSV-specific siRNA swarm derived from the sequence of bacterial lac repressor gene and referred here as a nonspecific control [8]. Additionally, a known immunostimulatory and cytotoxic long dsRNA, representing a sequence from a bacteriophage ϕ6 S segment, designated as 88 bp dsRNA, was used as a reference [18]. The enzymatic synthesis of the unmodified and modified siRNA swarms is described in more detail by Romanovskaya et al. and Levanova et al. [5,8]. All the produced RNAs were purified using monolithic chromatography [19].

**Table 1:** dsRNAs used in this study.

Short name of RNA	Type	RNA target sequence	Modification		Reference
			nt	%	
10% F-A	siRNA swarm	UL29 gene of HSV, 653 bp sequence	A	10	[8]
100% F-A			A	100	
unmodified			None		[7]
nonspecific		lac repressor gene of pET32b vector, 401 bp sequence	None		[8]
transgene specific		GFP gene of pCR3.1GFP plasmid [20], 717 bp sequence	None		[7]
88 bp dsRNA	dsRNA	bacteriophage ϕ6 S segment, 88 bp sequence	None		[18]

### Transfection

The HCE cells were transfected with 5 pmol/well (50 nM) of siRNA swarms, with 1 pmol/well (10 nM) of 88 bp cytotoxic control dsRNA or with water (mock treatment) on 96-well plates. The transfection was done using Lipofectamine RNAiMAX (Thermo Fisher, cat. 13778075) according to the manufacturer’s protocol of forward transfection.

### Virus

The virus HSV-1(17+)Lox-P_mCMV_GFP (abbreviated as HSV-1-GFP), was originally received from prof. Beate Sodeik (MHH Hannover Medical School, Germany) [21,22]. The virus was propagated in Vero cells (CCL-81, ATCC) as previously described [5]. For the antiviral assay, the HCE cells were infected with HSV-1-GFP at 1000 plaque forming units (pfu) in 100 μl per well (approximately 0.3 pfu/cell) on 96-well plates at four hours post transfection (hpt), as described before [5–9]. To quantify viral release (shedding), the culture supernatant was collected to separate 96-well plates at 44 hours post infection (hpi) and placed in -80°C until determination of viral titer by plaque assay on Vero cells in 96-well plates.

### Quantitative reverse transcription PCR

Expression levels of interferon beta (*IFN-β*), lambda 1 (*IFN-λ1; IL-29*), interferon stimulated gene 54 (*ISG54*), human myxovirus resistance protein A (*MxA*), human myxovirus resistance protein B (*MxB*), toll-like receptor 3 (*TLR3*) and interferon kappa (*IFN-κ*) genes as well as viral U_S_1, U_L_29, and U_L_48 genes were determined by quantitative reverse transcription PCR (RT-qPCR). The samples for the analysis were collected at 8, 24, or 48 hours post transfection by removing the supernatant and covering the cells with TRIzol Reagent (Invitrogen, cat. 15596026) after which the plate was placed to -80°C. After thawing, the cells were detached from the plate by vigorous resuspension, and the total cellular RNA was extracted according to the manufacturer’s protocol. The RNA was treated with DNase (Thermo Fisher, cat. EN0521) and processed into complementary (c)DNA with RevertAid H Reverse Transcriptase (Thermo Fisher, cat. EP0441) and random hexamer primers (Thermo Fisher, cat. SO142) as before [5–7]. The cDNA amounts were quantified with primers (S1 Table) mixed with SYBR Green enzyme (Thermo Fisher, cat. K0253) using QIAGEN Rotor-Gene Q (2-Plex) with Rotor-Gene Q Software 2.3.1.49. All the data was normalized to housekeeping gene (*GAPDH*) expression.

The primer sequences used for RT-qPCR, including both original and previously published sequences, are listed in S1 Table. The primer pair specific quantity standards, used as qPCR calibrators, are listed in S2 Table.

### Statistical analysis

Statistical analysis was done using SPSS Statistics 26.0.0.0 (IBM) or with GraphPad Prism version 8.4.3 for Windows (GraphPad Software). For determination of statistical significance for all assays quantifying antiviral efficacy, Kruskal-Wallis non-parametric test for multiple comparisons followed by Dunn’s multiple comparisons tests were used. For determination of differences of cellular responses between control and any RNA treatment, the statistical significances were determined with pairwise comparisons using Mann-Whitney’s non-parametric U-test. The nonlinear sigmoidal dose-response curve was fit and analyzed with GraphPad Prism.

## Results

### Antiviral siRNAs reduce HSV-1 replication and gene expression in HCE cells

**Fig 1:**
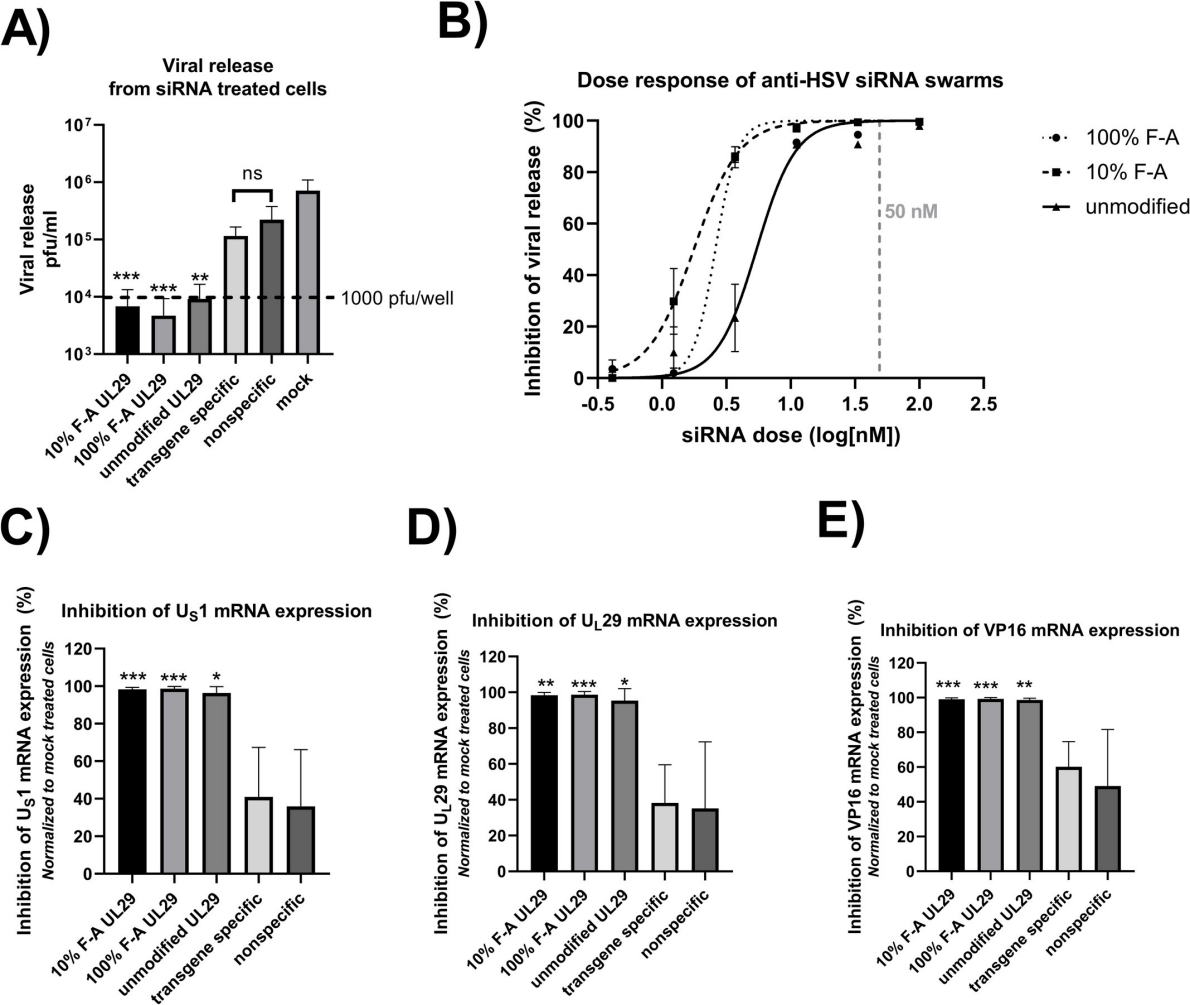
Antiviral activity of the siRNA swarms in human corneal epithelial cells. Human corneal epithelial (HCE) cells were transfected with the indicated siRNAs and infected with 1000 pfu of HSV-1-GFP four hours post transfection (hpt). At 48 hpt, samples were taken for the quantification of viral release (shedding) using plaque assay (**A** and **B**) and to measure viral mRNA expression (**C**–**E**). Both unmodified and modified anti-HSV UL29-siRNA swarms were used. The adenosine residues in the antisense strand of the 2’-fluoro modified siRNA swarms were either partially (referred to as 10% F-A) or fully (referred to as 100% F-A) modified. The controls in panels **A** and **C**–**E** include a transgene specific siRNA swarm, targeting the nonessential GFP insert of HSV-1-GFP, nonspecific siRNA swarm, targeting no gene present in the target cells nor in the virus, and mock treatment, referring to transfection reagent without any RNA. **(A)** Quantitation of viral release. HCE cells were treated with 5 pmols per well (50 nM) of the indicated siRNA swarms and infected 4 hpt with 1000 pfu of HSV-1-GFP per well. The column represents the mean and the whiskers the standard deviation of the treatment group. The data is from two independent experiments with a total of 8 replicates. The virus concentration representing the input virus amount of 1000 pfu/well in 100 μl, equaling to 10^4^ pfu/ml is indicated in the Fig 1A as a horizontal line (—). **(B)** Impact of siRNA dose on viral release. HCE cells were treated with a dose range of 0.41 to 100 nM (0.041 to 10 pmol per well, three-fold dilution series) of each indicated anti-HSV siRNA swarm and infected with 1000 pfu per well (96 well-plate) of HSV-1-GFP at 4 hpt. The error bars mark the standard error of mean (SEM) and the dashed line marks the 50 nM dose (5 pmols/well) used in all other panels. **(C-E)** HCE cells were treated with 50 nM of the indicated siRNA swarms and infected with 1000 pfu per well of HSV-1-GFP at 4 hpt. At 48 hpt, the cells were collected for quantitative reverse transcription PCR (RT-qPCR) analysis for the expression of three viral lytic genes, U_S_1, U_L_29, and U_L_48, encoding for the viral factors ICP22, ICP8, and VP16, respectively. The data is shown as percentage of the relative mRNA expression in comparison to the mock treated samples. The column represents the mean and the whiskers the standard deviation of the treatment group. The data is derived from two separate experiments, in which there were four biological replicates for each treatment group. In panels **A** and **C**-**E**, statistical significance is shown against mock treated samples (* p < 0.05, ** p < 0.01, *** p < 0.001).

The antiviral activity of unmodified UL29-targeted anti-HSV siRNA swarms and partially or fully 2’-fluoro-adenosine modified UL29-targeted anti-HSV siRNA swarms (10% F-A or 100% F-A, respectively) were studied in human corneal epithelial (HCE) cells (Fig 1). As control siRNA swarms, which do not have any target in actual genes of HSV-1, we used a transgene specific siRNA swarm, targeting the GFP marker insert of HSV-1-GFP, and a nonspecific siRNA swarm, targeting a sequence not expressed by the target cells nor by the virus. Additionally, we included a mock treatment control, referring to treatment with transfection reagent alone. For the antiviral assays, the cells were transfected with the antiviral RNA or controls, and at 4 hours post transfection (hpt) infected with 1000 plaque forming units (pfu) of HSV-1-GFP per well in 96-well plates.

At 44 hours post infection (hpi), the supernatants of HCE cells treated with 50 nM of 10% F-A, 100% F-A or unmodified UL29 siRNA swarms, or controls, were quantified for released virus by plaque assay (Fig 1A). All UL29 siRNA swarm treatments, whether done using partially, fully or non- 2’-fluoro modified siRNAs, led to a statistically significant decrease of viral release (p < 0.001, see Table 2 for exact p-values). Additionally, all UL29 siRNA swarms were capable of inhibiting the infection, as the mean of released virus from the UL29 siRNA swarm treated samples was less than the 1000 pfu dose used for infecting the cells (Fig 1A). The inhibition of viral release with unmodified, 10% F-A, and 100% F-A UL29 siRNA swarms was 98.7%, 99.0% and 99.3%, respectively, in comparison to the mock treatment (Table 2). The virus concentration representing the input virus amount of 1000 pfu/well in 100 μl, equaling to 10^4^ pfu/ml is indicated in the Fig 1A as a horizontal line (—).

**Table 2:** Antiviral activity of UL29 siRNA swarms in HCE cells^a^.

	IC_50_ (Fig 1B)	Inhibition of viral release (Fig 1A)			Inhibition of U_S_1 (ICP22) mRNA expression (Fig 1C)			Inhibition of U_L_29 (ICP8) mRNA expression (Fig 1D)			Inhibition of U_L_48 (VP16) mRNA expression (Fig 1E)		
	nM	Mean (%)	SEM (%)	p	Mean (%)	SEM (%)	p	Mean (%)	SEM (%)	p	Mean (%)	SEM (%)	p
**Unmodified**	5.43	98.7	0.006	0.0023	96.4	1.2	0.0165	95.4	2.3	0.0101	98.7	1.0	0.0030
**10% F-A**	1.77	99.0	0.006	0.0002	98.3	0.4	0.0009	98.3	1.6	0.0020	99.1	0.7	0.0002
**100% F-A**	2.6	99.3	0.004	<0.0001	98.7	0.4	0.0002	98.7	1.8	0.0001	99.2	0.7	<0.0001

^a^ The p values and inhibition % values are presented in comparison to mock treated samples. Except for the first column, the concentration of siRNA swarms was 50 nM. Details on the assay formats can be found in the respective figure legends, indicated in the column titles.

For determining the half-maximal inhibitory concentrations (IC_50_) of the anti-HSV UL29 siRNA swarms in HCE cells, the cells were transfected with 0.41 to 100 nM of the siRNA swarms 4 hours prior to infection (Fig 1B). The viral release was quantified 44 hpi from each sample. The IC_50_ –values for the unmodified, the 10% F-A and the 100% F-A UL29 siRNA swarms were 5.4, 1.8 and 2.6 nM, respectively (Fig 1B and Table 2). Hence, the 2’-fluoro-modified UL29 siRNA swarms have higher potency in HCE cells than unmodified siRNAs. The dashed line in the dose response curves (Fig 1B), denotes the 50 nM concentration of siRNA, which is used in all other assays as the concentration of the antiviral RNA.

To determine the inhibition of viral mRNA expression in response to antiviral siRNA swarm treatment, quantitation of mRNA levels of the U_S_1, U_L_29 and U_L_48 genes, which encode the viral factors ICP22, ICP8 and VP16, respectively, was conducted using RT-qPCR from samples taken at 48 hpt (44 hpi) (Fig 1C–1E). U_S_1, U_L_29 and U_L_48 represent alpha, beta, and gamma gene groups of HSV-1, respectively. All UL29 siRNA swarms reduced the viral gene expression by more than 95% (Fig 1C–1E, please see Table 2 for p-values). However, the different UL29 swarms did not show significant difference when compared to each other. This might partially reflect the excessive siRNA dose used in the experiment (i.e. doses onto the plateau of the dose-response curve; Fig 1B). Nevertheless, the tendency of the modified UL29-targeted siRNA swarms being more potent than the unmodified UL29-targeted siRNA swarms (Fig 1B and Table 2) in inhibition of the viral gene expression (Fig 1C–1E) as well as viral release (Fig 1A) was observed both in magnitude and as lower p-values (Table 2). Moreover, in contrast to the partially modified UL29 siRNA swarm (10% F-A), treatment with the fully modified UL29 siRNA swarm (100% F-A) led to higher inhibition of viral gene expression, of viral release, and altogether higher statistical significances (Table 2). Treatment with the non-HSV-specific siRNA swarms (the nonspecific and transgene specific siRNA swarms), did not differ significantly from the mock treatment in inhibition of viral release (Fig 1A) nor viral mRNA expression (Fig 1C–1E). Moreover, the nonspecific and transgene specific treatments did not differ when compared with each other (Fig 1C).

### Antiviral siRNAs reduce the accumulation rate of viral transcripts in HSV-1 infected HCE cells

**Fig 2:**
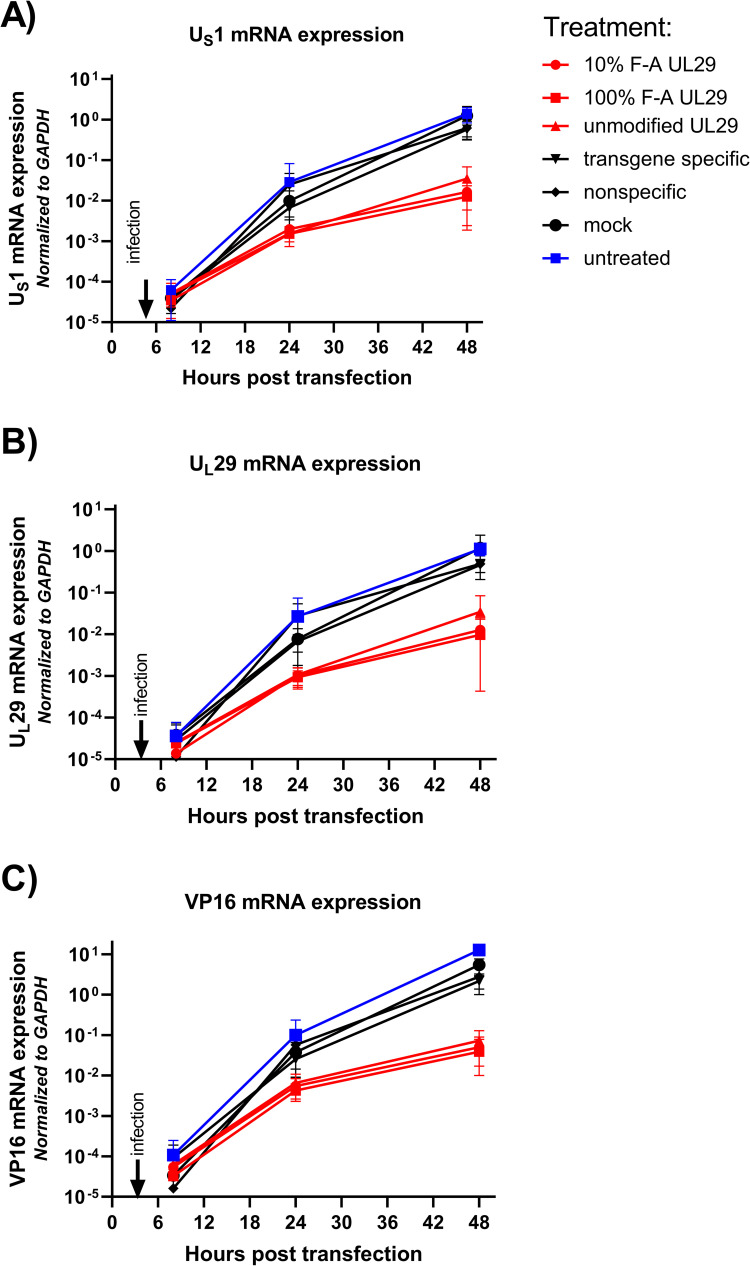
Kinetics of viral mRNA expression during antiviral RNAi treatment. HSV-1 infected HCE cells were transfected with the indicated siRNAs or controls at 50 nM in 96-well plates, and infected with 1000 pfu of HSV-1-GFP at 4 hours post transfection (hpt). The time point of infection is marked with an arrow in each of the panels. Samples were quantified with RT-qPCR for the expression of three viral genes, **(A)** U_S_1, **(B)** U_L_29, and **(C)** VP16 (U_L_48) at 6, 24, and 48 hpt. Viral mRNA expression levels of cells treated with UL29-targeted anti-HSV siRNA swarms (unmodified, 10% F-A, and 100% F-A) are in red, the untreated cells with blue, and the other control treatments in black. 10% F-A and 100% F-A are partially or fully 2’-fluoro-modified UL29 siRNA swarms, respectively. The control treatments include a transgene specific siRNA swarm, targeting the nonessential GFP gene insert of HSV-1-GFP, a nonspecific siRNA swarm, targeting a bacterial gene, and mock treatment, referring to transfection reagent without any RNA. The expression levels are normalized to housekeeping gene (*GAPDH*) expression. The data is derived from two separate experiments with at least four biological replicates for each treatment group in both experiments.

To explore the accumulation rates of viral transcripts in HCE cells, we analyzed how the treatment of cells with antiviral or control siRNAs, or mock treatment affect the accumulation of U_S_1, U_L_29, and U_L_48 mRNAs in HSV-1 infected HCE cells. We analyzed samples of treated (50 nM) and infected (1000 pfu/well) cell cultures with RT-qPCR at 8, 24, and 48 hpt (4, 20, and 44 hpi) (Fig 2).

The mRNA levels of all the studied viral genes increased in the HSV-1 infected HCE cells in the timespan of the experiment. Depending on the gene, the mRNA expression of the untreated infected HCE cells (marked with blue, in Fig 2) increased 100-fold (U_S_1 and U_L_29) or 1000-fold (U_L_48) between 4 hpi and 20 hpi, and 10-fold (U_S_1 and U_L_29) or 100-fold (U_L_48) between 20 and 44 hpi (Figs 2 and S1). The control treatment groups (marked with black, in Fig 2) followed the same profile.

At 8 hpt, the viral gene expression in infected cells, independent of the treatment group, was at an equal level. Subsequently, the viral gene expression increased more in the control groups than in cells treated with UL29 siRNA swarms (marked with black and red, respectively, in Fig 2) resulting in substantial differences in viral gene expression levels at 24 hpt, and at 48 hpt (Fig 2). All treatments with UL29 siRNA swarms led to very similar profiles of viral gene expression. Similarly, the viral gene expression profiles of the control treatments group together with the viral gene expression profiles of the untreated infected HCE cells. Altogether, the slope of change in viral gene expression in cells treated with different UL29 siRNA swarms is less steep between 0 and 24 hpt as well as between 24 and 48 hpt, than observed in the control treatments or untreated cells (Fig 2).

### Innate immunity responses of HCE cells to HSV-1 infection are slow and minor compared to those to immunostimulatory dsRNA

**Fig 3:**
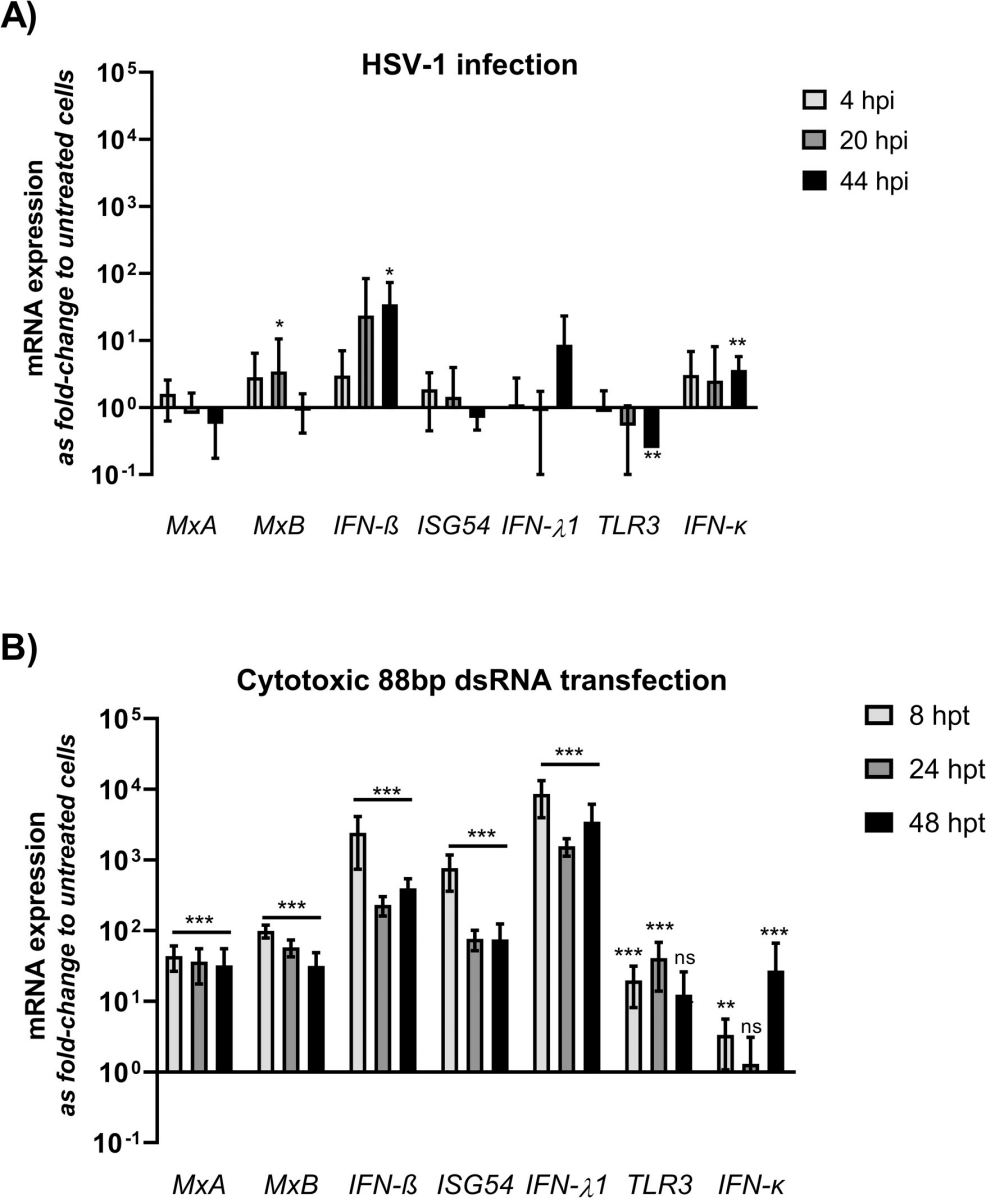
Relative innate immunity responses to HSV-1 infection and to cytotoxic 88bp dsRNA transfection in HCE cells. **(A)** HCE cells were infected with 1000 pfu of HSV-1-GFP per well on 96-well plates. At 4, 20, and 44 hours post infection (hpi) the cells were collected for RT-qPCR analysis of interferon beta (*IFN-β*), lambda 1 (*IFN-λ1; IL-29*), interferon stimulated gene 54 (*ISG54*), human myxovirus resistance protein A (*MxA*), human myxovirus resistance protein B (*MxB*), toll-like receptor 3 (*TLR3*) and interferon kappa (*IFN-κ*) mRNA expression. **(B)** HCE cells were transfected with 10 nM of the 88 bp dsRNA [18]. At 8, 24, and 48 hours post transfection (hpt) the cells were collected for RT-qPCR analysis *of IFN-β*, *IFN-λ1 (IL-29)*, *ISG54*, *MxA*, *MxB*, *TLR3* and *IFN-κ* mRNA expression. The data is normalized to housekeeping gene (GAPDH) expression and shown as fold-change to the expression levels of uninfected, untreated cells. The columns indicate the mean and the whiskers the standard deviation of the analyzed variable. The data is from two separate experiments with at least four biological replicates for each treatment group in both experiments. Statistical significance is shown against untreated, uninfected cells (* p < 0.05, ** p < 0.01, *** p < 0.001).

First, to evaluate how the innate immunity response profile of HCE cells is affected by HSV-1 infection, interferon beta (*IFN-β*), lambda 1 (*IFN-λ1; IL-29)*, interferon stimulated gene 54 (*ISG54*), human myxovirus resistance protein A (*MxA*), human myxovirus resistance protein B (*MxB*), toll-like receptor 3 (*TLR3*) and interferon kappa (*IFN-κ*) mRNA expression levels were quantified at 4, 20 and 44 hpi (Fig 3A). None of the studied markers was significantly different from uninfected control settings at 4 or 20 hours post infection. At 44 hpi, *IFN-β*, *IFN-κ* and *MxB* mRNA levels were significantly higher, and *TLR3* expression significantly lower, than those of uninfected, untreated cells. All other studied markers remained without significant change.

Next, to study whether HCE cells are able to mount a discernible innate immunity response to challenge with dsRNA, we studied type I and III interferon (*IFN*-β, *IFN*-κ, *IFN-λ1*), ISG (*ISG54*, *MxA*, *MxB*) and *TLR3* gene expression subsequent to transfection with the known immunostimulatory and cytotoxic dsRNA, the 88 bp dsRNA (Fig 3B). All studied mRNA levels, except for *TLR3* and *IFN-κ*, were significantly elevated in contrast to those of untreated cells at all time points. *TLR3* was significantly elevated only at the earlier time points, 8 hpt and 24 hpt, whereas *IFN-κ* was significantly elevated at 8 hpt and 48 hpt. Additionally, besides *IFN-κ*, all the studied mRNAs reached maximum induction at 8 hpt.

### HCE cells demonstrate similar innate immunity responses to antiviral siRNAs irrespective of HSV-1 infection status

To study innate immunity induction by siRNA swarms alone or in antiviral settings, the cells were left uninfected or were infected with 1000 pfu/well of HSV-1-GFP after transfection with the antiviral UL29 siRNA swarms (unmodified, 10% F-A, and 100% F-A), control swarms (the nonspecific and transgene specific siRNA swarms), or with mock treatment (water). The innate immunity responses, including type I and III interferon (*IFN-β*, *IFN-κ*, *IFN-λ1*), ISG (*ISG54*, *MxA*, *MxB*) and *TLR3* mRNA expression, were studied at 8, 24 and 48 hours post transfection, which equal to 4, 20 and 44 hours post infection (hpi), respectively.

**Fig 4:**
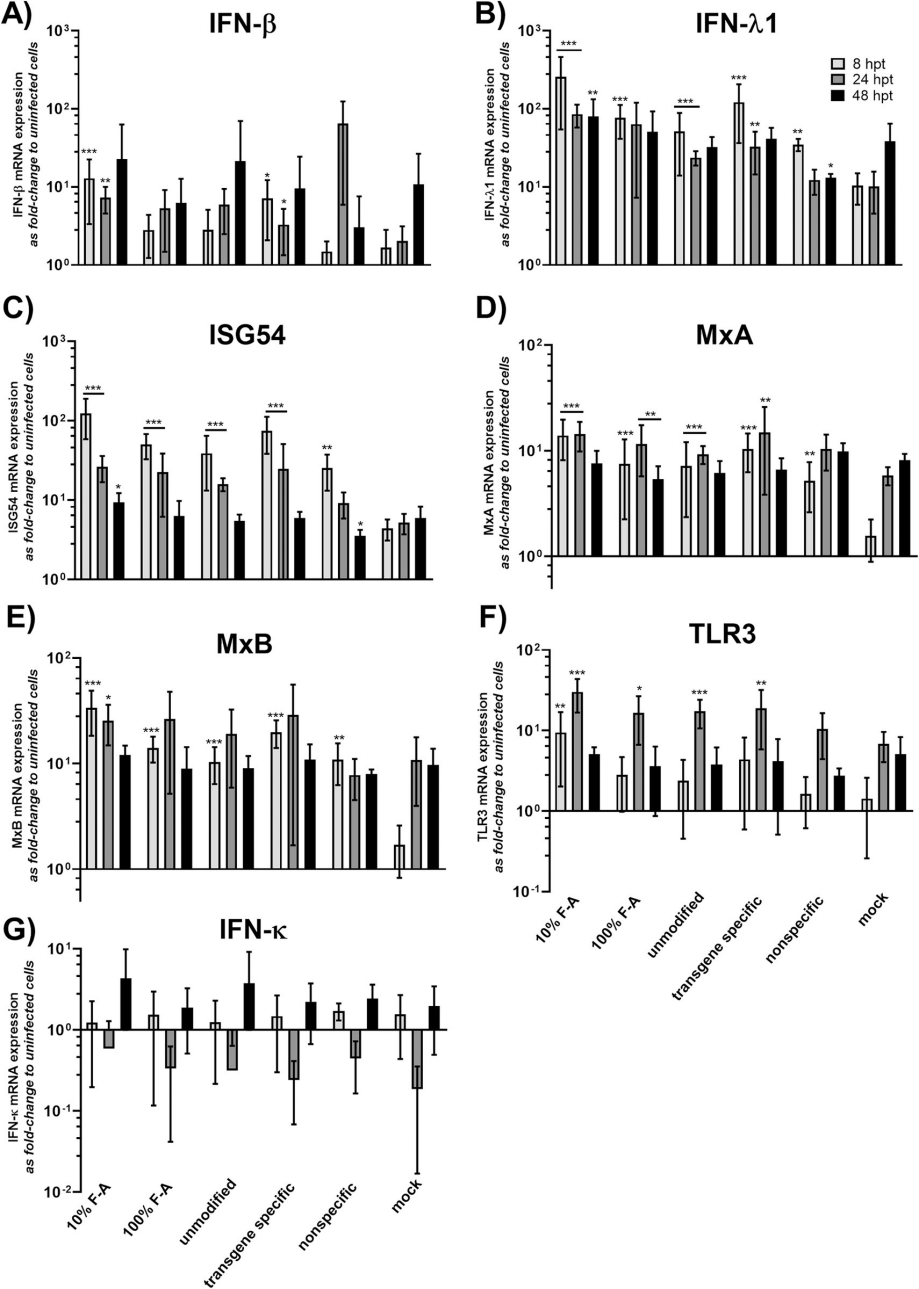
Innate immunity responses to the siRNA swarms. The levels of various innate responses of the HCE cells to siRNA swarms was determined by transfecting HCE cells in 96-well plates with the indicated siRNAs or controls at 50 nM. At 8, 24, and 48 hpt, the samples were quantified with RT-qPCR for the expression of **(A)** *IFN-β*, **(B)** *IFN-λ1 (IL-29)*, **(C)** *ISG54* **(D)** *MxA*, **(E)** *MxB*, **(F)** *TLR3* and **(G)** *IFN-к*. None of the siRNAs used have a specific target in this assay, as the cells were not infected. The unmodified, 10% F-A, and 100% F-A siRNA swarms all target the UL29 gene of HSV-1. 10% F-A and 100% F-A are modified UL29-siRNA swarms with partially or fully incorporated 2´-fluoro modified nucleotides, respectively. The control treatments, which do not have any target in wt HSV-1, include a transgene specific siRNA swarm, targeting the GFP insert of HSV-1-GFP, a nonspecific siRNA swarm, with no target in the cells nor in the virus, and mock treatment, referring to treatment with transfection reagent alone. All expression levels were normalized to housekeeping gene (GAPDH) expression and shown as fold change to uninfected, untreated cells. The columns indicate the mean and the whiskers the standard deviation of each treatment. The data is from two separate experiments with at least four biological replicates for each treatment group in both experiments. Statistical significance is shown against mock treated cells (* p < 0.05, ** p < 0.01, *** p < 0.001).

The responses of human corneal epithelial cells to siRNA swarms alone were altogether modest (Fig 4) and were consistently lower than those induced by the 88 bp reference dsRNA (Fig 3B). Furthermore, all treatments were well tolerated (S2 Fig). In comparison to the response induced by mock transfection, the inductions of *IFN-λ1*, *ISG54*, MxA and *MxB* were prominent, especially at the earliest time point. Notably, the 100% F-A modified siRNA swarm displayed more similar immunostimulation as the mock treatment, than the 10% F-A modified siRNA swarm did (Fig 4A, 4B, 4C, 4E and 4F).

**Fig 5:**
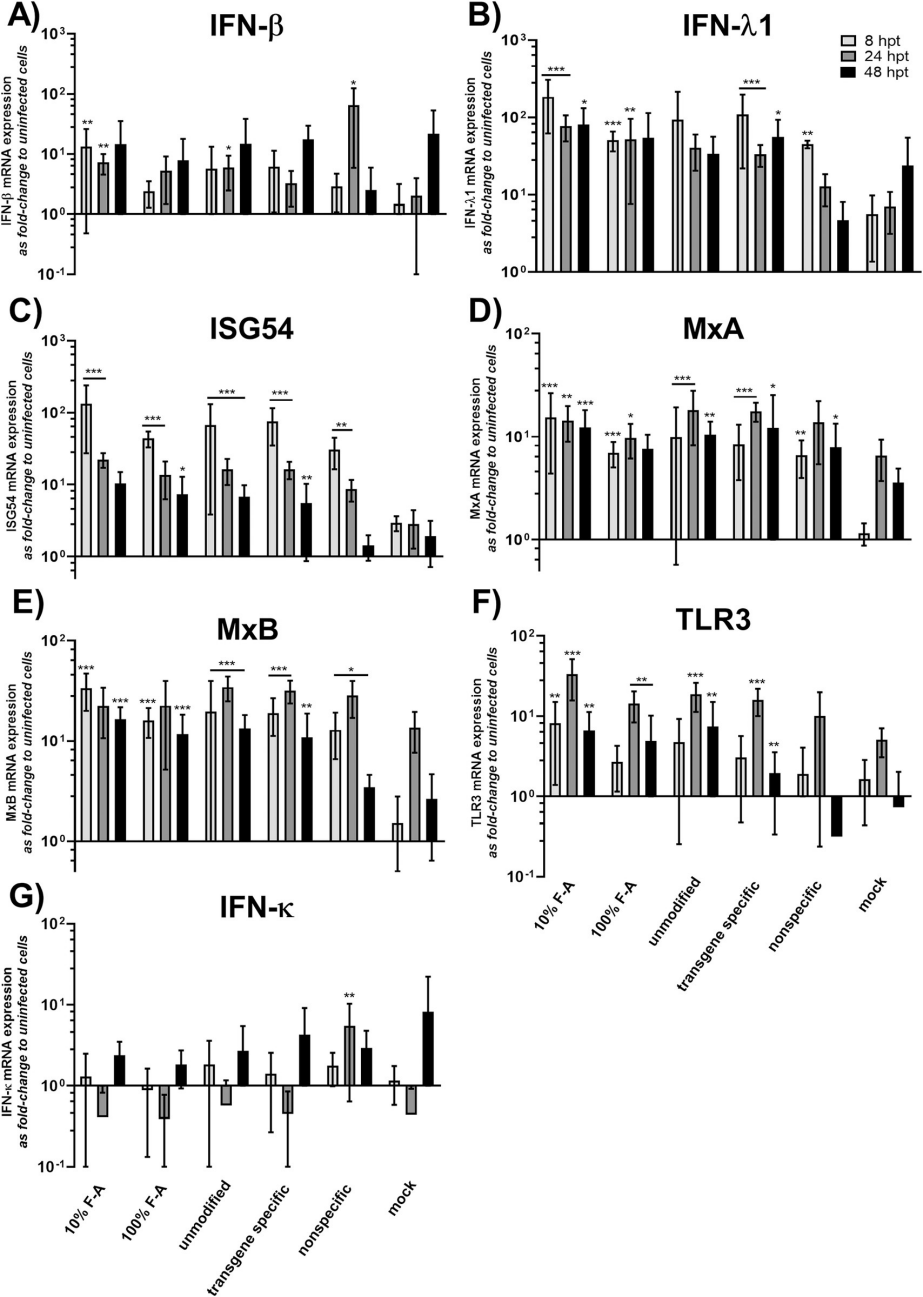
Innate immunity responses during antiviral siRNA swarm treatment of HSV-1 infected HCE cells. The innate immunity responses of HCE cells to siRNA swarms during an antiviral assay was determined by transfecting HCE cells with the indicated siRNAs or controls at 50 nM in 96-well plates and 4 hours post transfection (hpt) infecting the cells with 1000 pfu per well in 100 μl. At 8, 24, and 48 hpt (4, 20, and 44 hours post infection (hpi), respectively), the samples were quantified with RT-qPCR for the expression of **(A)** *IFN-β*, **(B)** *IL-29 (IFN-λ1)*, **(C)** *ISG54* **(D)** *MxA*, **(E)** *MxB*, **(F)** *TLR3* and **(G)** *IFN-к* mRNA expression. The unmodified, 10% F-A, and 100% F-A siRNA swarms all target the UL29 gene of HSV-1. 10% F-A and 100% F-A are modified UL29-siRNA swarms with partially or fully incorporated 2´-fluoro modified nucleotides, respectively. The control treatments, which do not have any target in wt HSV-1, include a GFP-specific siRNA swarm, targeting the nonessential GFP insert of HSV-1-GFP, a nonspecific siRNA swarm, targeting no gene expressed in the target cells nor the in virus, and mock treatment, referring to treatment with transfection reagent alone. The expression levels are normalized to housekeeping gene (GAPDH) expression and shown as fold change to uninfected, untreated cells. The columns indicate the mean and the whiskers the standard deviation of the indicated treatment. The data is from two separate experiments with at least four biological replicates for each treatment group in both experiments. Statistical significance is shown against mock treated cells (* p < 0.05, ** p < 0.01, *** p < 0.001).

The responses of human corneal epithelial cells to HSV-1 when pretreated with siRNAs (Fig 5), were very similar to the responses elicited by siRNAs alone (Fig 4). We observed similar profiles, similar statistical differences to mock treatment, and a similar magnitude of induction, when comparing the responses to siRNAs alone with responses to siRNAs with subsequent viral challenge (Figs 4 and 5, respectively). However, unlike in the cells treated with siRNAs alone, at the latest time point of the treatments involving virus infection, the mRNA levels of *MxB* (Fig 5E) and *TLR3* (Fig 5F) were significantly different from mock treatment, when subjected to the antiviral siRNAs or GFP-specific siRNA. However, whereas treatment with nonspecific dsRNA led to elevated *MxB* levels (Fig 5E), *TLR3* was not different in contrast to mock treatment (Fig 5F).

## Discussion

### The antiviral UL29 siRNA swarms were highly effective in HCE cells

HCE cells were responsive to antiviral treatment with UL29 siRNA swarms (Fig 1) on similar manner as has been shown before for other epithelial cell types [6,7,9]. Similarly to the previous observations, the UL29 siRNAs were antiviral in a sequence-specific manner, as cells treated with the non-HSV-specific siRNA swarms did not significantly differ from the mock treated cells neither by viral release nor by viral gene expression (Figs 1A, 1C, 1E and 2). In addition, the HSV transgene-specific siRNA did not induce a cellular responses capable of inhibiting virus, emphasizing sequence specificity, rather than a dsRNA-induced response in general. Furthermore, the innate immunity responses to all of the used siRNA swarms were modest (Figs 3B and 4).

In general, the UL29 siRNA swarms showed high antiviral potency against HSV-1 in HCE cells. Not only did they significantly decrease viral release by 99%, with more than 98% efficacy in downregulating the expression of the viral UL29 target gene, but they also decreased the observed net expression of the non-targeted viral α- and γ-genes, by more than 95% (Fig 1 and Table 2), as a result of limiting the replicating virus in the cell culture wells. Moreover, all UL29 siRNA swarms prevented the amount of released virus to surpass the viral inoculum (Fig 1A), proving that the UL29 siRNA swarms are capable of inhibiting HSV-1 infection for at least 44 hours upon prophylactic treatment. The efficacy of targeting UL29 is highlighted in the viral gene expression profiles, where all three UL29 siRNA swarms differ from the controls already at 24 hours post transfection (hpt) (Fig 2). Furthermore, the lower slope of the UL29 siRNA swarms in comparison to that of the controls between 24 and 48 hpt suggests that antiviral activity is not only due to prevention of the initial viral challenge, but that the siRNA hinders replication of progeny viruses as well. In future studies, it would be of high interest to further uncover whether the UL29 siRNA swarms remain active in treated cells and whether HSV can emerge from the treated culture at time points later than those studied in this research article. We anticipate that the modified UL29 siRNA swarms would be superior to unmodified siRNA swarms in this aspect, as the modified siRNA swarms have already demonstrated increased stability over unmodified siRNA swarms in presence of RNase A [8].

Previously, in cells of the nervous system, we demonstrated improvement of antiviral efficacy when 2´-fluoro modifications were incorporated into the UL29 siRNA swarms [8]. Likewise, with HCE cells, we evidenced elevation in antiviral efficacy when using modified siRNA swarms in contrast to using unmodified siRNA swarms: the modified UL29 siRNA swarms had higher antiviral potency (Fig 1B), higher percentages of inhibition of viral release (Fig 1A) and of viral gene expression (Fig 1C–1E), and higher statistical significance in their antiviral efficacy (Table 2), than the unmodified siRNA swarms. Furthermore, as before with cells of the nervous system [8], in HCE cells the partly modified UL29 siRNA swarm was less inhibitory and at lower statistical significances than the fully modified UL29 siRNA swarm (Fig 1 and Table 2). The results indicate a tendency of increased antiviral efficacy in HCE cells when using modified siRNA swarms in contrast to using unmodified siRNA swarms, and when using fully modified siRNA swarms in contrast to partly modified.

### The HCE cell line was able to mount antiviral responses, although the infection-induced innate immunity responses were low

The HCE cell line was permissive to our HSV-1-GFP marker virus infection, and suitable for antiviral RNAi study at the viral doses used, since we observed accumulation of replication competent progeny viruses to culture supernatant (Fig 1A and 1B) and expression of the viral genes U_S_1, U_L_29, and U_L_48 (S1 Fig) representing different regulatory gene groups. The expression levels of all studied viral genes increased throughout the experiment. The increase in expression was 10-fold higher from 4 hpi to 20 hpi in contrast to that from 20 hpi to 44 hpi (S1 Fig), reflecting efficient spread and replication of HSV-1 in HCE cells especially on the first day after the viral challenge. The setting with the low infectious dose mimicked a clinical infection or an *in vivo* reactivation situation, where the infection starts spreading from a few cells to many, rather the virus being in all the cells at the same time.

To study the innate immunity responses of HCE cells to HSV-1 infection, we analyzed infected HCE cells for mRNA expression of type I interferons (*IFN-β*, *IFN-κ*), a number of interferon stimulated genes (ISGs) (*MxA*, *MxB*, *ISG54*), a type III interferon (*IFN-λ1*), and a toll-like receptor (*TLR3*) (Fig 3A). The mRNA levels of *MxB*, *IFN-β*, *TLR3*, and *IFN-κ* were significantly influenced by HSV-1 infection (Fig 3A). However, these changes were all detected only at 44 hpi. The relatively low and late innate immunity response to prevalent viral replication (Figs 1A and S1) reflects the fact that HSV-1 is capable of modulating many or all of the studied innate immunity responses in HCE cells. The capability of HSV-1 in immune evasion is evidenced by the detected downregulation of TLR3 (Fig 3A), reported previously to be modulated by the viral U_S_3 protein kinase [23]. In addition to potentially *de novo* expressed HSV immunoevasion genes, several HSV tegument proteins, capable of modulating antiviral responses, enter the cells with the incoming stock virus and have likely influenced the observed siRNA-induced antiviral signaling. Certainly, indirect downregulation of the studied markers due to modulation of an upstream function is likely. Nevertheless, at 44 hpi, HCE cells were able to induce significantly increased expression of *IFN-β*, *IFN-κ* and *MxB* to counteract the infection (Fig 3A), which might have in part hindered the increase in viral gene expression from 20 to 44 hpi (S1 Fig) as *IFN-β*, *IFN-κ* and *MxB* are antiviral [24,25].

### Cytotoxic 88bp dsRNA evoked strong immune responses and toxicity in HCE cells

Transfection with the phage ϕ6-derived 88 bp dsRNA has been cytotoxic and induced high innate immunity responses in the cell lines tested before [5,8,18], which allowed 88 bp dsRNA to be used as a positive reference for cellular toxicity. Hence, we wanted to elucidate, whether 88 bp dsRNA is immunostimulatory in HCE cells as well. Indeed, transfection with 88 bp dsRNA was cytotoxic (S2 Fig) in HCE cells, and markedly upregulated the expression of the studied type I and III interferons (*IFN-β*, *IFN-λ1*) and all ISGs (MxA, *MxB*, *ISG54*) at all studied time points, with the tendency of peaking at the earliest time point (Fig 3B). *IFN-κ* and *TLR3* expression were not as consistently induced as were the others (Fig 3B). However, all the studied markers were inducible in HCE cells and the induction was successful by 88 bp dsRNA. The extent of the 88 bp dsRNA induction was higher than that by HSV-1 (Fig 3) in all studied innate immunity expression levels, except with *IFN-κ* whose expression was induced to equal levels by HSV1 and 88 bp dsRNA. Due to the strong evoked immune response and toxicity, 88 bp is highly antiviral in HCE cells (S3 Fig).

### Innate immunity responses of HCE cells to antiviral RNA are modest, regardless of the viral challenge

For treatment of corneal HSV infection with RNAi, it is of high importance to develop antiviral RNA with non-cytotoxic innate immunity responses, as the inflammatory balance of the cornea is delicate. Slight induction of type I or type III innate responses might favor the antiviral activity of the RNA, but overly extensive induction might be toxic to the target tissue. In the previously studied cell types, siRNA swarms induce only slight type I and type III innate responses [6,8]. Here, we studied HCE cells for mRNA expression of type I interferons (*IFN-β*, *IFN-κ*) and various interferon stimulated genes (ISGs) (*MxA*, *MxB*, *ISG54*), as well as type III interferon (*IFN-λ1*) and toll-like receptor (*TLR3*) after transfection with antiviral UL29 siRNA swarms, or controls. As before [8], the induction was modest, most prevalent at the earliest time point and mostly related to the cells response to transfection rather than the dsRNA (Fig 4). The mRNA levels of any of the studied markers did not reach those induced by the 88 bp dsRNA, confirming that the response to the delivery of dsRNA to HCE cells is not toxic. In comparison to the response induced by mock transfection, the inductions of *IFN-λ1*, *ISG54*, *MxA* and *MxB* were most prominent, especially at the earliest time point. Although there was no statistical difference between the 10% F-A and 100% F-A modified siRNA swarms, the 100% F-A modified siRNA swarm was less different from mock treatment than the 10% F-A modified siRNA swarm in a majority of the responses studied (Fig 4A, 4B, 4C, 4E and 4F). This finding of decreased tendency for immunostimulation by 100% F-A modified siRNA swarm in contrast to 10% F-A modified siRNA swarm is in line with our previous results, derived from a cell type representing the nervous system [8]. Furthermore, as the interplay of HSV-1 and the dsRNA may be complicated due to immune evasion of the host responses by the virus (Fig 3, [26]) and shared pathways of pattern recognition, we studied the same responses during antiviral treatment in infected cells (Fig 5). The innate responses of HCE cells in the antiviral setting were mostly similar to those of dsRNA alone. Interestingly, presence of an RNAi target in the virus did not have any effect on the innate response of HCE cells, as all dsRNAs had similar induction profiles independent of infection. The effect of HSV-1 infection to the innate responses was evident only in case of the *TLR3* expression (Fig 5F), which was decreased below the level of the baseline cellular expression in the treatment groups with the most prevalent HSV-1 infection (Fig 1A), as expected (Fig 3A).

## Conclusion

We have previously shown the effect of antiviral siRNA swarms against HSV in retinal, epithelial and neuronal cells. In the current study, we showed the antiviral efficacy also in cells of corneal epithelium, human corneal epithelial cells (HCE), which to our knowledge have not before been used for antiviral RNAi studies of HSV infection. We observed that HCE cells do support our HSV-GFP infection, and display type I innate responses, including *IFN-β*, *IFN-κ* and *MxB* induction. We also showed that in HCE various type I and III innate immunity responses are induced by a known immunostimulatory dsRNA. The induced innate responses to our antiviral siRNA swarms were only modest, and mostly similar to those induced by the transfection reagent alone. The antiviral siRNA harboring 2´-fluoro modifications were similarly, or better, tolerated than the unmodified antiviral siRNA, and displayed slightly higher antiviral efficacy, especially when 100% of the adenosines in the sequence were modified. In the antiviral assay, the innate responses remained modest, indicating safety for use of dsRNA-mediated antiviral RNAi in human cornea. As a conclusion, the HCE cells are an appropriate, translational cell line for determining the efficacy and tolerability of RNAi for HSV infection *in vitro*. In this cell type, the 2´-fluoro adenosine modified siRNA swarms were highly antiviral and well tolerated, which encourages for further research on antiviral siRNA swarm therapy of infected cornea, especially with modified siRNA swarms.

## Supporting information

S1 Fig Expression of viral mRNA in untreated HSV-1 infected HCE cells. HCE cells were infected with 1000 pfu of HSV-1-GFP per well on 96-well plates. At 4, 20, and 44 hours post infection samples were collected and analyzed by RT-qPCR using primers specific for the viral genes US1, UL29, and UL48. The data is from two independent experiments with at least four replicates each, and is normalized to housekeeping gene (GAPDH) expression.
(PDF)

S2 Fig Cytotoxicity of modified siRNA swarms to HCE cells. HCE cells were transfected with 50 nM of the indicated treatments and measured for cellular viability at 48 hpt with CellTiterGlo (Promega, Madison, WI) as described in Levanova et al.. The modified siRNA swarms tested had either a fraction (10%) or all (100%) of adenosine (F-A), cytidine (F-C) or uridine (F-U) nucleotides 2’-fluoro-modified. Additionally, lipofectamine alone, a non-specific siRNA swarm and a cytotoxic 88bp RNA were included for comparisons. Cellular viability of the treatments is presented as relative viability to untreated samples. The asterisks indicate significant difference in cellular viability compared to the treatment with the lipofectamine alone (* p ≤ 0.05, ** p ≤ 0.01, *** p ≤ 0.001). The bars represent the mean and the whiskers the standard deviation of the mean (N≥8 per treatment, data from two individual experiments).
(PDF)

S3 Fig The cytotoxic 88 bp RNA reduces viral gene expression. Human corneal epithelial (HCE) cells were transfected with the 88 bp dsRNAs (10 nM), unmodified UL29 siRNA swarm (50 nM), nonspecific siRNA swarm (50 nM) or lipofectamine transfection reagent alone (mock treatment) and infected with 1000 pfu of HSV-1-GFP four hours post transfection (hpt). At 48 hpt, samples were quantified for viral mRNA expression. At 48 hpt, the cells were collected for quantitative reverse transcriptase PCR (RT-qPCR) analysis for the expression of three viral genes, (A) US1, (B) UL29, and (C) UL48, encoding ICP22, ICP8, and VP16, respectively. The data is from two separate experiments with at least 4 replicates in each. The data is shown as mRNA expression normalized to housekeeping gene (GAPDH) expression. The columns represent the mean and the whiskers the standard deviation of the treatment group. The statistical significance is shown against mock treated (“Lipofectamine”) samples (*** p < 0.001).
(PDF)

S1 Table Primers used for RT-qPCR. (PDF)

S2 Table Quantitative PCR quantity standards. Quantity standards (calibrators), specifically custom-made for each primer pair, were used in RT-qPCR analysis for quantification. The standards were amplified from cDNA of human cells with the indicated primers, purified and used in the qPCR-run as quantity standards with dilutions of 10^8^ to 100 copies per reaction.
(PDF)
