# Supplementary material for: Swarms of chemically modified antiviral siRNA targeting herpes simplex virus infection in human corneal epithelial cells
Source: PLoS Pathog. 2022 Jul 6;18(7):e1010688. doi: 10.1371/journal.ppat.1010688 (PMC9292126; doi:10.1371/journal.ppat.1010688)
Supplement: S1 Fig — HCE cells were infected with 1000 pfu of HSV-1-GFP per well on 96-well plates. At 4, 20, and 44 hours post infection samples were collected and analyzed by RT-qPCR using primers specific for the viral genes US1, UL29, and UL48. The data is from two independent experiments with at least four replicates each, and is normalized to housekeeping gene (GAPDH) expression. (PDF) [file ppat.1010688.s001.pdf]

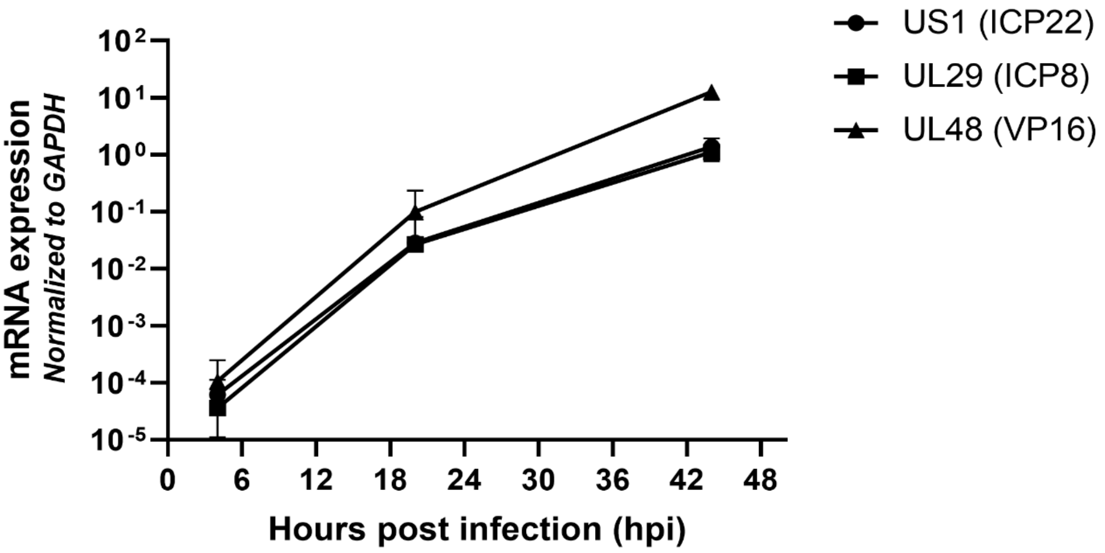

Supplementary Figure 1. Expression of viral mRNA in untreated HSV-1 infected HCE cells. HCE cells were infected with 1000 pfu of HSV-1-GFP per well on 96-well plates. At 4, 20, and 44 hours post infection samples were collected and analyzed by RT-qPCR using primers specific for the viral genes U_S1, U_L29, and U_L48. The data is from two independent experiments with at least four replicates each, and is normalized to housekeeping gene (GAPDH) expression.
