# Supplementary material for: Swarms of chemically modified antiviral siRNA targeting herpes simplex virus infection in human corneal epithelial cells
Source: PLoS Pathog. 2022 Jul 6;18(7):e1010688. doi: 10.1371/journal.ppat.1010688 (PMC9292126; doi:10.1371/journal.ppat.1010688)
Supplement: S2 Fig — HCE cells were transfected with 50 nM of the indicated treatments and measured for cellular viability at 48 hpt with CellTiterGlo (Promega, Madison, WI) as described in Levanova et al.. The modified siRNA swarms tested had either a fraction (10%) or all (100%) of adenosine (F-A), cytidine (F-C) or uridine (F-U) nucleotides 2’-fluoro-modified. Additionally, lipofectamine alone, a non-specific siRNA swarm and a cytotoxic 88bp RNA were included for comparisons. Cellular viability of the treatments is presented as relative viability to untreated samples. The asterisks indicate significant difference in cellular viability compared to the treatment with the lipofectamine alone (* p ≤ 0.05, ** p ≤ 0.01, *** p ≤ 0.001). The bars represent the mean and the whiskers the standard deviation of the mean (N≥8 per treatment, data from two individual experiments). (PDF) [file ppat.1010688.s002.pdf]

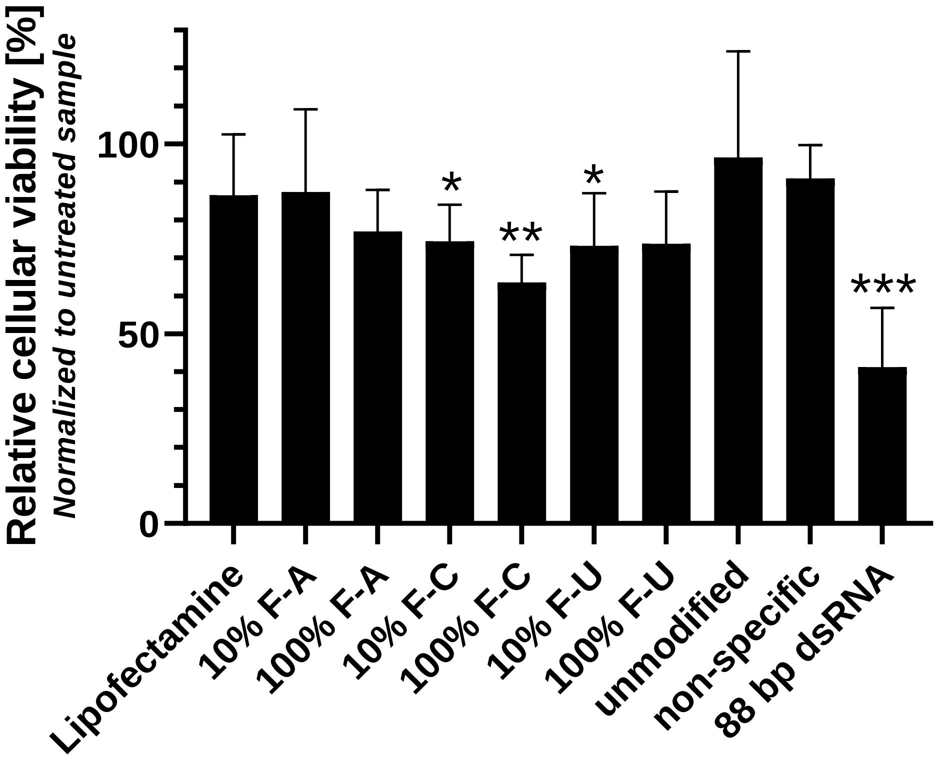

Supplementary figure 2. Cytotoxicity of modified siRNA swarms to HCE cells. HCE cells were transfected with 50 nM of the indicated treatments and measured for cellular viability at 48 hpt with CellTiterGlo (Promega, Madison, WI) as described in [1]. The modified siRNA swarms tested had either a fraction (10%) or all (100%) of adenosine (F-A), cytidine (F-C) or uridine (F-U) nucleotides 2'-fluoro-modified. Additionally, lipofectamine alone, a non-specific siRNA swarm and a cytotoxic 88bp RNA were included for comparisons. Cellular viability of the treatments is presented as relative viability to untreated samples. The asterisks indicate significant difference in cellular viability compared to the treatment with the lipofectamine alone (* $p \leq 0.05$, ** $p \leq 0.01$, *** $p \leq 0.001$). The bars represent the mean and the whiskers the standard deviation of the mean ($N \geq 8$ per treatment, data from two individual experiments).

References

1. Levanova AA, Kalke KM, Lund LM, Sipari N, Sadeghi M, Nyman MC, et al. Enzymatically synthesized 2'-fluoro-modified Dicer-substrate siRNA swarms against herpes simplex virus demonstrate enhanced antiviral efficacy and low cytotoxicity. Antiviral Res. 2020;182:104916. Epub 2020/08/18. doi: 10.1016/j.antiviral.2020.104916. PubMed PMID: 32798603; PubMed Central PMCID: PMCPMC7424292.
