# Supplementary material for: Swarms of chemically modified antiviral siRNA targeting herpes simplex virus infection in human corneal epithelial cells
Source: PLoS Pathog. 2022 Jul 6;18(7):e1010688. doi: 10.1371/journal.ppat.1010688 (PMC9292126; doi:10.1371/journal.ppat.1010688)
Supplement: S3 Fig — Human corneal epithelial (HCE) cells were transfected with the 88 bp dsRNAs (10 nM), unmodified UL29 siRNA swarm (50 nM), nonspecific siRNA swarm (50 nM) or lipofectamine transfection reagent alone (mock treatment) and infected with 1000 pfu of HSV-1-GFP four hours post transfection (hpt). At 48 hpt, samples were quantified for viral mRNA expression. At 48 hpt, the cells were collected for quantitative reverse transcriptase PCR (RT-qPCR) analysis for the expression of three viral genes, (A) US1, (B) UL29, and (C) UL48, encoding ICP22, ICP8, and VP16, respectively. The data is from two separate experiments with at least 4 replicates in each. The data is shown as mRNA expression normalized to housekeeping gene (GAPDH) expression. The columns represent the mean and the whiskers the standard deviation of the treatment group. The statistical significance is shown against mock treated (“Lipofectamine”) samples (*** p < 0.001). (PDF) [file ppat.1010688.s003.pdf]

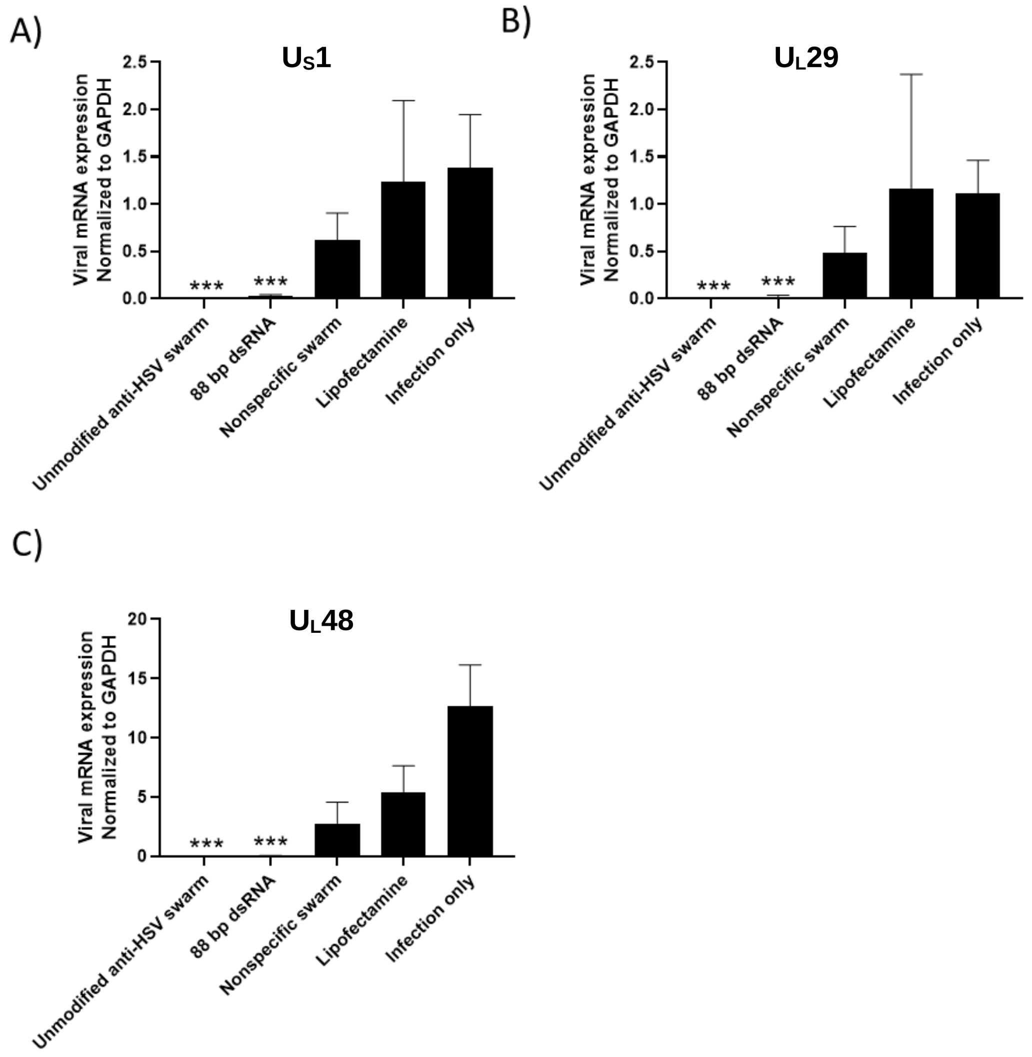

Supplementary Figure 3. Cytotoxic 88 bp RNA reduces viral gene expression. Human corneal epithelial (HCE) cells were transfected with the 88 bp dsRNAs (10 nM), unmodified UL29 siRNA swarm (50 nM), nonspecific siRNA swarm (50 nM) or lipofectamine transfection reagent alone (mock treatment) and infected with 1000 pfu of HSV-1-GFP four hours post transfection (hpt). At 48 hpt, samples were quantified for viral mRNA expression. At 48 hpt, the cells were collected for quantitative reverse transcriptase PCR (RT-qPCR) analysis for the expression of three viral genes, **(A)** U_S1, **(B)** U_L29, and **(C)** U_L48, encoding ICP22, ICP8, and VP16, respectively. The data is from two separate experiments with at least 4 replicates in each. The data is shown as mRNA expression normalized to housekeeping gene (GAPDH) expression. The columns represent the mean and the whiskers the standard deviation of the treatment group. The statistical significance is shown against mock treated (“Lipofectamine”) samples (*** $p < 0.001$).
