# Supplementary material for: Swarms of chemically modified antiviral siRNA targeting herpes simplex virus infection in human corneal epithelial cells
Source: PLoS Pathog. 2022 Jul 6;18(7):e1010688. doi: 10.1371/journal.ppat.1010688 (PMC9292126; doi:10.1371/journal.ppat.1010688)
Supplement: S1 Table — (PDF) [file ppat.1010688.s004.pdf]

S1 Table. Primers used for RT-qPCR.

Target		Sequence	Reference
GAPDH[a]	sense	GAG AAG GCT GGG GCT CAT	[1]
	antisense	TGC TGA TGA TCT TGA GGC TG	
IFN-β[b]	sense	TCT CCA CGA CAG CTC TTT CCA	[2]
	antisense	ACA CTG ACA ATT GCT GCT TCT TTG	
IL-29[b]	sense	GAC GCC TTG GAA GAG TCA C	[3]
	antisense	CTC ACC TGG AGA AGC CTC A	
ISG54[a]	sense	ACT ATC ACA TGG GCC GAC TC	[4]
	antisense	TTT AAC CGT GTC CAC CCT TC	
MxA[b]	sense	GAG GAG ATC TTT CAG CAC CTG	[5]; This study
	antisense	TGG ATG ATC AAA GGG ATG TG	
MxB[a]	sense	GGA AAG CAG CGT CCT TCT CT	This study
	antisense	ATT CCT TCC AGC AAC AGC CA	
TLR3[a]	sense	TAG CAG TCA TCC AAC AGA ATC AT	[2]
	antisense	AAT CTT CTG AGT TGA TTA TGG GTA A	
IFN-κ[b]	sense	CAG AAA CTC TTG GGG CAA CTC	This study
	antisense	TCA CCT GAG AAG AGT CAC CTG	
**U_S1**[b]	sense	CAT GCG CCA GTG TAT CAA TC	[3]
	antisense	CGG CAG TAT CCC ATC AGG TA	
**U_L29**[b]	sense	AAG CTG GTT GCG TTG GAG	[4]
	antisense	TTT CTG CTG AAG CAG TTC CA	
**U_L48**[b]	sense	TTT GAC CCG CGA GAT CCT AT	[6]
	antisense	GCT CCG TTG ACG AAC ATG AA	

[a] Annealing temperature of 55°C in the PCR cycle, [b] Annealing temperature of 60°C in the PCR cycle

References

1. Nygardas M, Vuorinen T, Aalto AP, Bamford DH, Hukkanen V. Inhibition of coxsackievirus B3 and related enteroviruses by antiviral short interfering RNA pools produced using phi6 RNA-dependent RNA polymerase. J Gen Virol. 2009;90(Pt 10):2468-73. Epub 2009/06/26. doi: 10.1099/vir.0.011338-0. PubMed PMID: 19553393.
2. Peri P, Mattila RK, Kantola H, Broberg E, Karttunen HS, Waris M, et al. Herpes simplex virus type 1 Us3 gene deletion influences toll-like receptor responses in cultured monocytic cells. Virol J. 2008;5:140. Epub 2008/11/26. doi: 10.1186/1743-422X-5-140. PubMed PMID: 19025601; PubMed Central PMCID: PMCPMC2605447.
3. Paavilainen H, Lehtinen J, Romanovskaya A, Nygardas M, Bamford DH, Poranen MM, et al. Inhibition of clinical pathogenic herpes simplex virus 1 strains with enzymatically created siRNA pools. J Med Virol. 2016;88(12):2196-205. Epub 2016/05/19. doi: 10.1002/jmv.24578. PubMed PMID: 27191509.
4. Romanovskaya A, Paavilainen H, Nygardas M, Bamford DH, Hukkanen V, Poranen MM. Enzymatically produced pools of canonical and Dicer-substrate siRNA molecules display comparable gene silencing and antiviral activities against herpes simplex virus. PLoS One. 2012;7(11):e51019. Epub 2012/12/12. doi: 10.1371/journal.pone.0051019. PubMed PMID: 23226452; PubMed Central PMCID: PMCPMC3511422.
5. Yahya M, Rulli M, Toivonen L, Waris M, Peltola V. Detection of Host Response to Viral Respiratory Infection by Measurement of Messenger RNA for MxA, TRIM21, and Viperin in Nasal Swabs. J Infect Dis. 2017;216(9):1099-103. doi: 10.1093/infdis/jix458. PubMed PMID: 28968760.

6. Broberg EK, Nygardas M, Salmi AA, Hukkanen V. Low copy number detection of herpes simplex virus type 1 mRNA and mouse Th1 type cytokine mRNAs by Light Cycler quantitative real-time PCR. J Virol Methods. 2003;112(1-2):53-65. Epub 2003/09/03. doi: 10.1016/s0166-0934(03)00191-5. PubMed PMID: 12951213.
