# Supplementary material for: Swarms of chemically modified antiviral siRNA targeting herpes simplex virus infection in human corneal epithelial cells
Source: PLoS Pathog. 2022 Jul 6;18(7):e1010688. doi: 10.1371/journal.ppat.1010688 (PMC9292126; doi:10.1371/journal.ppat.1010688)
Supplement: S2 Table — Quantity standards (calibrators), specifically custom-made for each primer pair, were used in RT-qPCR analysis for quantification. The standards were amplified from cDNA of human cells with the indicated primers, purified and used in the qPCR-run as quantity standards with dilutions of 108 to 100 copies per reaction. (PDF) [file ppat.1010688.s005.pdf]

S2 Table. Quantitative PCR quantity standards. Quantity standards (calibrators), specifically custom-made for each primer pair, were used in RT-qPCR analysis for quantification. The standards were amplified from cDNA of human cells with the indicated primers, purified and used in the qPCR-run as quantity standards with dilutions of 10^8 to 10^0 copies per reaction.

Target	Primer pair	
GAPDH	forward<br>reverse	AAT CCC ATC ACC ATC TTC CA<br>TGA GTC CTT CCA CGA TAC CA
IFN-β	sense<br>antisense	AGA CTG CTC ATG CGT TTT CC<br>TCC TCC AAA TTG CTC TCC TG
ISG54	sense<br>antisense	AAG CCA CAA TGT GCA ACC AT<br>GAG CCT TCT CAA AGC ACA CC
MxB	sense<br>antisense	CTG AAC GTG CAG CGA GCT T<br>TCG ATG AGG TCA ATG CAG GG
TLR3	sense<br>antisense	ATG AAA TGT CTG GAT TTG GAC TA<br>GTT AGC TGG CTA TAC CTT GTG A
**U_S1**	sense<br>antisense	AAG CCC AAA TGC AAT GCT AC<br>CAG ACA CTT GCG GTC TTC TG
**U_L29**	sense<br>antisense	GGT GCG GTC AAA AAT AAG GA<br>CCT ACC AGA AGC CCG ACA AG
**U_L48[1]**	-	
***IL-29*[1]**	-	
***MxA*[1]**	-	
***IFN-κ*[1]**	-	

[1]The quantity standards (calibrators) of qPCR assays for interleukin 29 (IFN $\lambda 1$), MxA, interferon kappa, and HSV UL48 mRNAs were pUNO1-hIL29 (InvivoGen), a cDNA clone of human MxA (used in [1]), pUNO1-hIFNK (InvivoGen), and the plasmid clone pRB3717 [2], respectively.

References

1. Yahya M, Rulli M, Toivonen L, Waris M, Peltola V. Detection of Host Response to Viral Respiratory Infection by Measurement of Messenger RNA for MxA, TRIM21, and Viperin in Nasal Swabs. J Infect Dis. 2017;216(9):1099-103. doi: 10.1093/infdis/jix458. PubMed PMID: 28968760.
2. McKnight JL, Kristie TM, Roizman B. Binding of the virion protein mediating alpha gene induction in herpes simplex virus 1-infected cells to its cis site requires cellular proteins. Proc Natl Acad Sci U S A. 1987;84(20):7061-5. doi: 10.1073/pnas.84.20.7061. PubMed PMID: 2823252; PubMed Central PMCID: PMCPMC299229.
